# Cost-Effectiveness of Highly Active Antiretroviral Therapy in South Africa

**DOI:** 10.1371/journal.pmed.0030004

**Published:** 2005-12-06

**Authors:** Motasim Badri, Gary Maartens, Sundhiya Mandalia, Linda-Gail Bekker, John R Penrod, Robert W Platt, Robin Wood, Eduard J Beck

**Affiliations:** **1**Department of Medicine, Desmond Tutu HIV Centre, University of Cape Town, Cape Town, South Africa; **2**Department of Clinical Pharmacology, University of Cape Town, Cape Town, South Africa; **3**NPMS-HHC Coordination and Analytic Centre, Chelsea and Westminster Hospital, London, United Kingdom; **4**Department of Medicine, Imperial College, Chelsea and Westminster Hospital, London, United Kingdom; **5**Division of Clinical Epidemiology, McGill University Health Centre, Montreal, Quebec, Canada; **6**Department of Epidemiology and Biostatistics, McGill University, Montreal, Quebec, Canada; St. Vincent's HospitalAustralia

## Abstract

**Background:**

Little information exists on the impact of highly active antiretroviral therapy (HAART) on health-care provision in South Africa despite increasing scale-up of access to HAART and gradual reduction in HAART prices.

**Methods and Findings:**

Use and cost of services for 265 HIV-infected adults without AIDS (World Health Organization [WHO] stage 1, 2, or 3) and 27 with AIDS (WHO stage 4) receiving HAART between 1995 and 2000 in Cape Town were compared with HIV-infected controls matched for baseline WHO stage, CD4 count, age, and socioeconomic status, who did not receive antiretroviral therapy (ART; No-ART group). Costs of service provision (January 2004 prices, US$1 = 7.6 Rand) included local unit costs, and two scenarios for HAART prices for WHO recommended first-line regimens: scenario 1 used current South African public-sector ART drug prices of $730 per patient-year (PPY), whereas scenario 2 was based on the anticipated public-sector price for locally manufactured drug of $181 PPY. All analyses are presented in terms of patients without AIDS and patients with AIDS.

For patients without AIDS, the mean number of inpatient days PPY was 1.08 (95% confidence interval [CI]: 0.97–1.19) for the HAART group versus 3.73 (95% CI: 3.55–3.97) for the No-ART group, and 8.71 (95% CI: 8.40–9.03) versus 4.35 (95% CI: 4.12–5.61), respectively, for mean number of outpatient visits PPY. Average service provision PPY was $950 for the No-ART group versus $1,342 and $793 PPY for the HAART group for scenario 1 and 2, respectively, whereas the incremental cost per life-year gained (LYG) was $1,622 for scenario 1 and $675 for scenario 2. For patients with AIDS, mean inpatients days PPY was 2.04 (95% CI: 1.63–2.52) for the HAART versus 15.36 (95% CI: 13.97–16.85) for the No-ART group. Mean outpatient visits PPY was 7.62 (95% CI: 6.81–8.49) compared with 6.60 (95% CI: 5.69–7.62) respectively. Average service provision PPY was $3,520 for the No-ART group versus $1,513 and $964 for the HAART group for scenario 1 and 2, respectively, whereas the incremental cost per LYG was cost saving for both scenarios. In a sensitivity analysis based on the lower (25%) and upper (75%) interquartile range survival percentiles, the incremental cost per LYG ranged from $1,557 to $1,772 for the group without AIDS and from cost saving to $111 for patients with AIDS.

**Conclusion:**

HAART is a cost-effective intervention in South Africa, and cost saving when HAART prices are further reduced. Our estimates, however, were based on direct costs, and as such the actual cost saving might have been underestimated if indirect costs were also included.

## Abstract 2

Healthcare costs for HIV-infected South African adults on HAART compared with costs for HIV-infected controls not on HAART. Authors conclude HAART is cost effective.

## Introduction

South Africa is experiencing an HIV epidemic with enormous social and economic consequences. Recent estimates suggest that between 4.5 and 6.2 million of the 43 million South Africans are infected with HIV-1 [1]. There were 370,000 AIDS deaths during 2003 [1], and the cumulative projected AIDS mortality for 2010 is 4–7 million in absence of a highly active antiretroviral therapy (HAART) programme [2]. The largest impact of HIV on the public health sector lies in the hospital sector [3]. In the year 2000, HIV-related admissions amounted to 24% of all public hospital admissions [4] and 12.5% of the total public health budget [5]. Cost of inpatient and ambulatory health care of both private and public health-care sectors is expected to rise rapidly [5].

The cost-effectiveness of HAART, in terms of reducing HIV-related morbidity and mortality, has been documented in industrialized countries [6–12]. The introduction of combination HAART into routine clinical care in these countries has been associated with a shift from inpatient to outpatient-based hospital care [11–17]. Until recently the prevailing assumption was that the public sector of the South African health-care system was unable to afford the introduction of antiretroviral therapy (ART) in routine clinical care. However, the government of South Africa recently announced its commitment towards creating the necessary conditions for introducing ART into the public health sector [18]. In addition, the price of HAART for resource-poor countries decreased markedly since the year 2000 [19,20]. The South African Department of Health has recently awarded contracts for the supply of ART drugs to public health facilities countrywide to international pharmaceutical companies [21]. This tender is expected to reduce HAART price to $181 per patient-year (PPY).

The aim of this study was to compare use and cost of HIV-1–related service provision between patients receiving HAART and a comparison group not receiving ART, and assess the cost effectiveness of HAART.

## Methods

### Study Population

This study was based on the Cape Town AIDS Cohort ( CTAC); a prospective cohort study which has been described previously [22,23]. In brief, patients of this cohort were accrued from the HIV clinics affiliated to the University of Cape Town, who were referred from a wide range of primary HIV health-care providers. During the study period 1st January 1995 to 31st December 2000, HAART was not available in the publicly funded South African health-care sector. All patients in this study accessed HAART through the participation in the international HAART multicentre phase III clinical trials, as approved by the Research Ethics Committee of the University of Cape Town.

For the purpose of this study, all patients who participated in the clinical trials and received at least three ART drugs—a non-nucleoside reverse transcriptase inhibitor or protease inhibitor together with two nucleoside analogues or three nucleoside analogues—were included as the treated arm of the study (HAART group). Patients were excluded from the clinical trials if they were active injecting-drug users, were diagnosed with an acute opportunistic infection at the time of recruitment, were reported to have significant laboratory abnormalities, or if they were treated with immune-modulating or systemic chemotherapeutic agents. Lactating or pregnant women were also excluded. The trial visit schedule was usually at weeks 2, 4, and 8 and then every two to three months thereafter.

Patients who did not participate in these clinical trials and never had access to ART throughout the study period (No-ART group) but received other HIV-related care were the sample from which a “comparator” group was identified for the HAART group.

At each clinic visit, all patients were routinely examined for HIV related manifestations and staged using the World Health Organization (WHO) clinical HIV staging system [24]. HIV-1 infection was diagnosed by enzyme-linked immunosorbent assay (ELISA) tests and confirmed by Western blot or a second enzyme-linked immunosorbent assay test. Viral load (which was available only for the HAART group) was determined by reverse transcriptase-polymerase chain reaction (Amplicor; Roche Molecular Systems, Branchburg, New Jersey, United States) and CD4^+^ count, measured by flow cytometry (Beckman Coulter, Miami, Florida, United States).

### Analysis

This analysis calculated the use and cost of HIV service provision and compared the clinical outcome, in terms of disease progression or life year gained (LYG) by clinical stage of HIV infection, between patients receiving HAART and a matched comparison group who did not receive ART (No-ART group). Patients were classified as either being non-AIDS (WHO stages 1, 2, or 3) or AIDS (WHO stage 4) patients.

Several strategies were employed to ensure that the two groups studied were clinically, immunologically, and socioeconomically similar and matched for the same variables used to recruit the HAART group into the clinical trials. Logistic regression models were fitted to identify factors associated with receiving HAART in this cohort using SAS GENMODE procedure with logit link function and binomial error distribution [25]. HAART patients were individually matched with randomly selected No-ART patients on the basis of variables independently associated with the likelihood of receiving HAART. The socioeconomic status of each patient was classified into “low” or “high”, using a composite index developed by the Cape Metropolitan Council [26]. A subgroup logistic regression analysis was performed for the HAART group, to examine whether the likelihood of hospitalisation differed by HAART class.

To examine for residual confounding, the matched case–control data were analysed using a conditional logistic regression model, stratified by matching variables. The model was fitted using the SAS PHREG procedure with discrete logistic model. All data analyses were performed in SAS version 8.02. χ^2^ was used to compare categorical variables, and the non-parametric median test was used to compare continuous non-normally distributed variables. All *p-*values quoted are two sided, with a *p-*value < 0.05 considered as significant.

### Use and Cost of Services

Information on inpatient and outpatient care was obtained from the computerized hospital information systems supplemented by case notes. The mean number of inpatient days and outpatient visits PPY were calculated for the non-AIDS (WHO stage 1, 2, and 3) and AIDS (WHO stage 4) WHO clinical stages for both HAART and No-ART groups. A patient-year was defined as 365.25 days of follow up and methods used for calculating the mean use of services were similar to those used in other studies [12,16,17,27]. The denominator consisted of the total duration of follow up for all patients seen during the study period and numerators were calculated by summing the use of each service. Mean and 95% confidence intervals (CI) of inpatient and outpatient service use PPY by WHO stage were calculated for the two groups using the binomial distribution, and were compared between the two groups by calculating the odds ratio (OR) of the use of inpatient and outpatient services, using the No-ART group as a reference group.

The costs of hospital HIV service provision were calculated from a public health-care system perspective [28–30]. Unit costs were obtained from a detailedcosting study of HIV inpatient and outpatient care conducted in the year 2000 [31], and were adjusted for inflation to financial year 2004 prices using the South African Consumer Price Index [32]. Prices were converted from South African Rand to US dollars using the average exchange rate for 2004 (US dollars = 7.6 Rand) [33]. The unit cost was $215 for an inpatient day and $33 for an outpatient visit and included costs for tests including CD4 counts, procedures, and non-ART drug costs. The non-ART drugs included all drugs other than ART dispensed to the patients during the course of care, including treatment and prophylaxis for opportunistic infections. Mean inpatient days and outpatient visits PPY were multiplied by their respective unit costs to estimate the PPY cost of service provision.

ART prices used in this study are those currently available to the public health-care sector (Ministry of Health, Provincial Administration of the Western Cape). HAART drug-price scenarios presented were (1) present public sector prices, which amounted to $730 per annum, and (2) anticipated public sector price for locally manufactured drugs, which amounted to $181 per annum, for the WHO-recommended regimen for resource-limited settings [34].

To estimate the total cost of service provision PPY for HAART patients for the two scenarios, average ART drug costs PPY were added to the average inpatient and outpatient PPY costs. In sensitivity analysis, minimum and maximum ART drug PPY costs for the two scenarios were also added to the lower and upper limit of the 95% CI: inpatient and outpatient PPY cost of care to provide a range of costs. Viral load was not measured for the No-ART group because it was not available in publicly funded institutions during the study period and, therefore, PPY cost of viral-load investigation of $79 (D. Roditti, personal communication) was only added to the annual cost of service provision for the HAART group.

### Cost of LYG by WHO Stage of HIV Infection

Progression times were calculated from date of entry into non-AIDS (WHO stage 1, 2, or 3) to date of progression to AIDS (WHO stage 4) or death, and from initial diagnosis of AIDS (WHO stage 4) to death for AIDS patients. Patients not known to have progressed during follow-up were censored at either the most recent visit to the clinic or when lost to follow-up. Median progression times were estimated using the product-limit Kaplan-Meier survival method, and these were compared for the HAART and No-ART groups using log-rank test. Due to the small number of individuals who progressed during the follow-up period, median and interquartile ranges (IQR) for time to progression to AIDS or death were extrapolated from the product-limit time to failure estimates using the maximum likelihood least squares method. The progression-free times for non-AIDS and AIDS patients for each group were multiplied by the average PPY cost of service provision, and the additional life years gained of non-AIDS and AIDS groups was calculated as the incremental cost per LYG, based on the difference in the estimated median progression times of the two groups [27].

Because discounting health benefits remains controversial [35], only non-discounted estimates are presented. However, given the relatively short time in each WHO stage, it is unlikely that an analysis with a non-zero discount rate would yield qualitatively different results than those presented here.

### Sensitivity Analysis

Robustness of results was assessed in a sensitivity analysis; accounting for variances associated with treatment effects and total cost of service provision. IQRs between the lower (25%) and upper (75%) progression-free times percentiles of the non-AIDS and AIDS patients were multiplied by the average and 95% CI of the cost of service provision, and the incremental cost per LYG was calculated.

## Results

### Study Sample

Of the 1,630 patients in the cohort, 292 patients (265 non-AIDS and 27 with AIDS) received HAART through participation in the clinical trials. The rest of the patients (*n* = 1,328; 1,093 non-AIDS and 235 with AIDS) did not have access to ART during the study period and comprised the population from which the No-ART comparator group for the 292 patients who received HAART was identified. Baseline CD4 count, WHO stage, age, and socioeconomic status were independently associated with the likelihood of receiving HAART (Table 1), but gender was not, and therefore this variable was not considered in further analyses. Matching was therefore based on WHO stage, CD4 count (<200, 200–350, and >350 cells/μl), age (less than the median age or equal to the median age or greater of the non-AIDS and AIDS groups respectively) and socioeconomic status (low or high socioeconomic status).

**Table 1:**
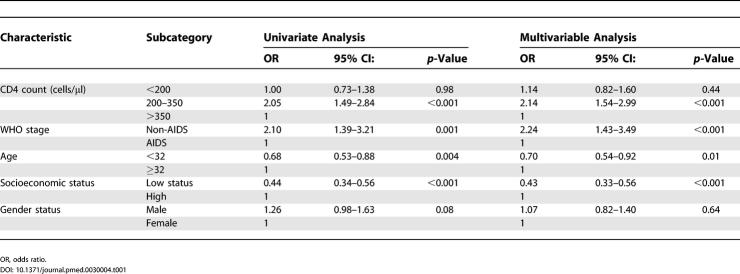
Univariate and Multivariate Logistic Regression Analyses of Factors Associated with the Likelihood of Receiving HAART

HAART drug classes were not independently associated with increased risk of hospitalisation (Table 2) and were therefore analysed as one category. The characteristics of the final study population of the 292 patients who received HAART and the 292 matched No-ART patients are described in Table 3.

**Table 2:**
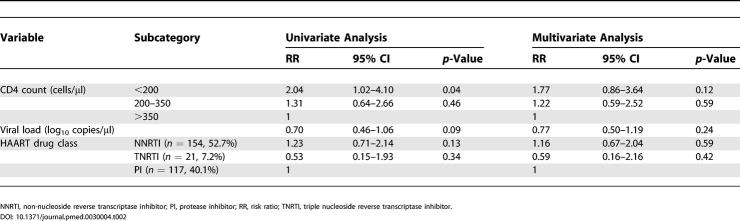
Logistic Regression Analysis of Factors Associated with Hospitalisation among the Treated Group

**Table 3:**
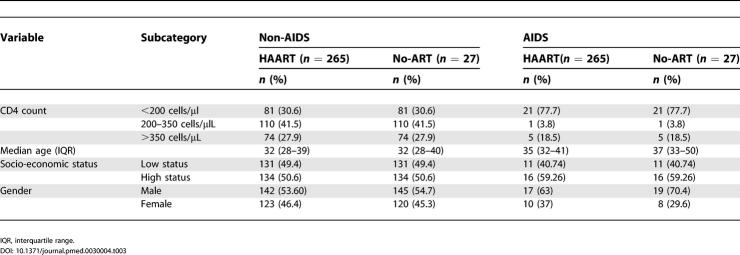
Baseline Demographic and Clinical Characteristics of the Matched Non-AIDS and AIDS Groups

### The Non-AIDS Population (WHO Stage 1, 2, or 3)

The matched non-AIDS group included 265 patients both in the HAART and No-ART group. Approximately one-third of the patients in the two groups had a baseline CD4 count <200 cell/μl and (49.4%) were of low socioeconomic status. Median age at inclusion into study did not differ in the two groups; 32 y, [IQR: 28–39 y] in the HAART group versus 32 y [IQR: 28–40 y] in the No-ART group (median test *p* = 0.48). Although not matched for, gender distribution did not differ statistically in the two groups (χ^2^ = 0.07, *p* = 0.79; Table 3). Median progression time was significantly longer in the HAART group compared with the No-ART group at 4.1 and 3.0 y respectively (log-rank test χ^2^ = 36.6, *p* < 0.001; Figure 1).

**Figure 1:**
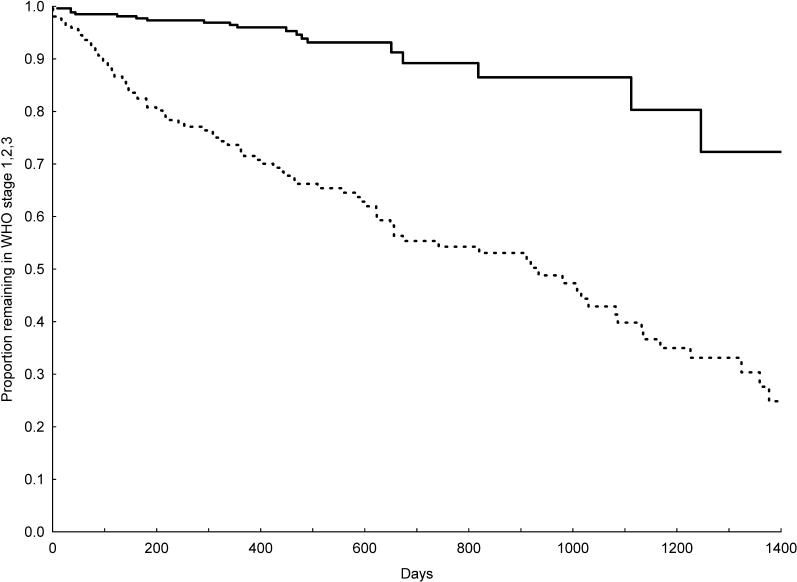
Progression of HIV-Infected Individuals from Non-AIDS Stages (WHO Stage 1, 2, or 3) for Patients on HAART and Not on ART The solid line indicates patients on HAART, and the dotted line indicates patients not on ART.

### Use and Cost of Services and Cost per LYG

Patients on HAART had 1.08 (95% CI: 0.97–1.19) mean inpatient days, significantly fewer than the 3.75 d (95% CI: 3.55–3.97) of the No-ART group; χ^2^ = 147, OR = 0.29, 95% CI: 0.23–0.36, *p* < 0.001; but had significantly more outpatient visits of 8.71 (95% CI: 8.40–9.03) compared with 4.35 (95% CI: 4.12–5.61); χ^2^ = 145, OR = 2.00, 95% CI: 1.78–2.25, *p* < 0.0001 (Table 4). The average PPY inpatient cost in the HAART group was significantly less than that for the No-ART group, while the average costs of outpatient visits PPY for the No-ART group were less than those for the HAART group (Table 4).

**Table 4:**
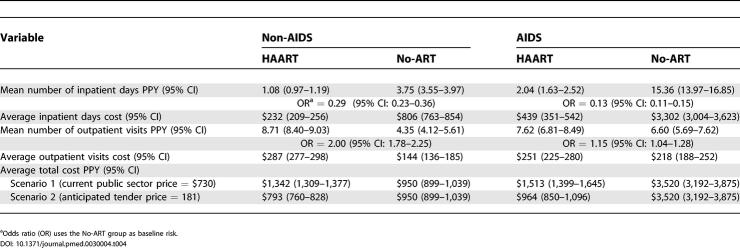
Mean Number of Inpatient Days, Outpatient Visits, and Associated Cost^a^ PPY

Based on the two HAART price scenarios, the average cost of service provision PPY for the HAART group ranged from a minimum of $760 to $1,377 PPY, with scenario 2 having the lowest service provision cost (Table 4). The incremental cost per LYG for median progression time was $1,622 (95% CI: 1,607–1,627) for scenario 1 and $675 (95% CI: 659–679) for scenario 2 (Table 5). When a sensitivity analysis was performed based on the IQR of the progression times, the incremental cost per LYG varied between $1,578 (95% CI: 1,557–1,581) and $1,759 (95% CI: 1,748–1,772) for the 25th and 75th percentiles respectively (Table 5).

**Table 5:**
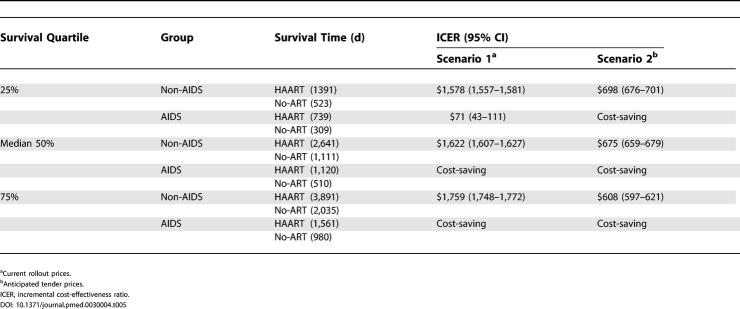
Incremental Cost-Effectiveness Ratio (US$) for Current ART Rollout Prices (US$730 Per Annum—Scenario 1) and Anticipated Tender Prices (US$181 Per Annum—Scenario 2), Comparing HAART and No-ART Groups for Non-AIDS and AIDS Groups at 25th, 50th (Median), and 75th Progression-Free Times Percentiles

### The AIDS Population (WHO Stage 4)

The AIDS population included 27 patients in each group. The majority of patients in the two groups presented with a CD4 count <200 cell/μl (77%), and 40.74% were of low socioeconomic status. Median age did not differ in the two groups; 35 y (IQR: 32–41) in the HAART group versus 37 y (IQR: 33–50) in the No-ART group (median test *p* = 0.27). Gender distribution, with 63% and 70.4% males in the HAART and No-ART groups respectively, was not significantly different in the two groups (χ^2^ = 0.33, *p* = 0.56) (see Table 3). Median progression time was significantly longer in the HAART group compared with the No-ART group; at 3.1 and 1.4 y respectively (log-rank χ^2^ = 5.28, *p* = 0.02; Figure 2).

**Figure 2:**
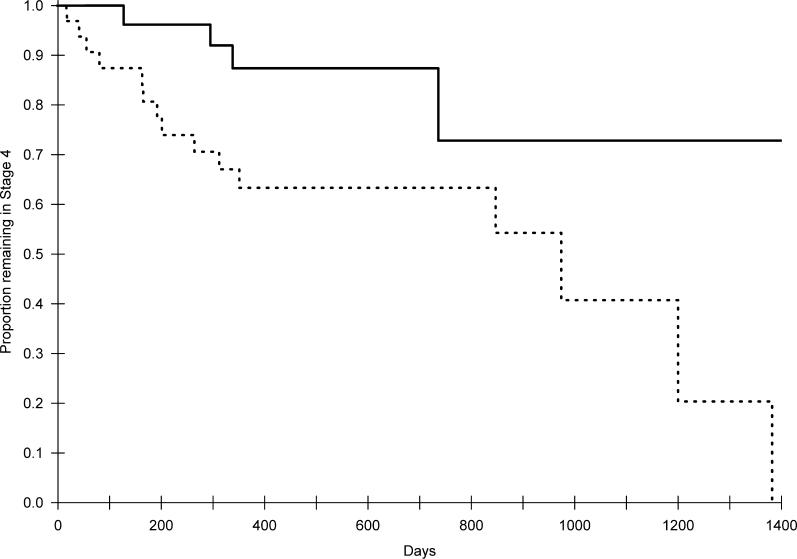
Progression of HIV-Infected Individuals from WHO Stage 4 for Patients on HAART and Not on ART The solid line indicates patients on HAART, and the dotted line indicates patients not on ART.

### Use and Cost of Services and Cost per LYG

Patients on HAART had significantly fewer mean PPY inpatient days at 2.04 d (95% CI: 1.63–2.52) compared with 15.36 d (95% CI: 13.97–16.85) for the No-ART group (χ^2^ = 1,019, OR = 0.13, 95 CI: 0.11–0.15, *p* < 0.0001). Mean outpatient visits PPY in the two groups did differ significantly; at 7.62 (95% CI: 6.81–8.49) for the HAART group compared with 6.60 (95% CI: 5.69–7.62) for the No-ART group; χ^2^ = 7.3, OR = 1.15, 95% CI: 1.04–1.28, *p* = 0.007, though not as substantially as for the non-AIDS group (see Table 4). The average inpatient cost PPY in the HAART group was significantly less than that for the No-ART group, but the average costs of outpatient visits PPY in the groups were not significantly (see Table 4).

Based on the two HAART price scenarios, the average cost of service provision PPY for the HAART group ranged from a minimum of $850 to $1,645 PPY, with the lowest care cost observed for scenario 2 (see Table 4). For patients diagnosed with AIDS, the incremental cost per LYG for the median progression time was cost saving for both HAART price scenarios (Table 5). When a sensitivity analysis was performed based on the IQR of the progression times, the incremental cost per LYG varied between $71 (95% CI: 43–111) and cost saving for the 25th and 75th progression-free time percentiles respectively (Table 5).

## Discussion

This study, employing methods used in similar studies from industrialized countries [27], provides a unique contemporaneous comparison of the use, cost, and outcome of hospital service provision for a group of HIV-infected patients in Cape Town receiving HAART compared with an immunologically, clinically, and socioeconomically similar group of patients who did not receive ART. Use of HAART was associated with decreased disease progression, AIDS, and death. The HAART group used fewer inpatient services than the No-ART group, and the magnitude of these changes did not differ by HAART regimens used in this study. The reduction in use of inpatient services, which has been observed in similar studies in industrialized countries [10–15], was most likely due to a reduction in morbidity and mortality [6,12]. The use of services increased for both groups with increased severity of HIV infection, resulting in an increased cost of service provision. The increased use of inpatient services for patients with AIDS is most likely related to AIDS-related events or their terminal phase of their illness [36–41]. In Zimbabwe, medical insurance claims of privately insured HIV-infected patients in the last few months of their lives were 700% higher than that of uninfected patients in the same age group [42].

To date, very few cost-effectiveness studies have been performed on HAART in a South African setting [43]. The incremental cost per LYG ranged from being cost saving to $1,772. The cut-off point for what constitutes a cost-effective intervention per outcome measure varies from society to another. For instance, the cost-effective cut-off point in the United States is currently considered to be $50,000 per outcome measure and £30,000 in the United Kingdom [30]. To date such a consensus on what would constitute a realistic threshold for South Africa has not yet emerged, but a cut-off of twice the per capita gross domestic product (GDP) has been suggested as a reasonable cut-off point for developing countries [44]. For the year 2004, the per capita gross domestic product in South Africa was $3,480, and therefore this threshold would amount to $6,960 [45]. The cost per LYG of two HAART cost scenarios for the non-AIDS and AIDS patients showed that introducing HAART in this hospital setting would be a very cost-effective intervention. However, it is clear that the cost-effectiveness ratios were very sensitive to the price of HAART. If prices of the awarded tender could be achieved, the introduction of HAART will be a very cost-effective intervention in Cape Town and probably in similar settings in sub-Saharan Africa, because HIV accounts for between 40% and 70% of the public sector inpatient service provision in the region [3,36–40].

Concern has been expressed that increased access to HAART in sub-Saharan Africa will result in the widespread viral resistance due to poor adherence [46]. Studies performed in a number of sub-Saharan African countries, however, have shown that the proportion of individuals maintaining viral suppression is comparable to that reported from developed countries [47–49].

This study did have a number of limitations. Because HAART was not used in routine clinical practice, we had to compare a group of patients enrolled in clinical trials with a control group that was not part of the trials. Individuals who take part in clinical trials have to fulfil certain entry criteria, as well as to conform to well-defined protocols and scheduled attendances. It is therefore difficult to exclude the possibility that a selection bias might have resulted from the inclusion/exclusion criteria of these clinical trials. However, the No-ART control group was selected on the basis of clinical, socioeconomic, and immunologic characteristics similar to those individuals recruited into the HAART trials conducted in this study. The frequency of inpatient and outpatient services utilization of the HAART and No-ART patients in this study is similar to that reported by UK and Canadian observational studies [12,27]. However, in this study, the sample is relatively small for the AIDS group.

This study was focused on hospital services provided at the level of a teaching hospital. Therefore the costs incurred through primary, community, or secondary hospital care were not included, but this reflected the configuration of services available to the majority of HIV-infected people in Cape Town at the time of the study. Similarly, the costs included were direct costs only and did not incorporate the indirect or intangible costs, such as loss of productivity or quality of life associated with this illness, because currently no such data exist in South Africa. Some studies from the United Kingdom have demonstrated that from a public sector perspective, indirect costs can comprise between 58% and 124% of direct treatment costs for HAART or between 45% and 102% from a societal perspective [30]. If these costs were all included, it is likely that the cost-effectiveness ratio would even be more favourable. Our estimates did not incorporate the costs of providing the infrastructure required to support appropriate HAART provision in rollout programmes. However the rollout programmes were designed to start from settings where infrastructure currently exists, which would predominantly be urban. Recent reports estimated that if the public sector included HAART as part of a package of HIV treatment and care in the year 2003, the cost would be 1.2% of the South African GNP, which is unlikely to push health-care expenditure beyond prudent levels [50,51].

The recent commitment towards scale-up of HAART in South Africa as part of HIV treatment and care has been an important and positive development. The urgent need to introduce HAART as part of routine HIV treatment and care was recently re-iterated in a World Bank report, which indicated that if this is not done soon, failure to do so would have devastating effects on this and future generations of South Africans [52].

Although the primary rationale for wider access to HAART is humanitarian, a national HAART programme targeting patients with symptomatic HIV disease, using low-cost HAART prices would also significantly decrease hospital services utilization by HIV-infected patients, resulting in either health expenditure saving by cost deferral or freeing substantial resources for health care of non-HIV patients.

**Patient Summary**

**Background**

The number of cases of AIDS continues to increase worldwide; the disease is a major threat to humanity, with Africa facing the very worst problems. In South Africa alone there were 370,000 AIDS deaths in 2003. AIDS is caused by a type of retrovirus—the human immunodeficiency virus (HIV). Highly active antiretroviral treatment (HAART) is a treatment that uses a combination of three or more antiretroviral drugs that attack different parts of the virus. HAART is expensive, making it difficult for poor countries to provide treatment for all who need it. Prices are falling, however, and South Africa is one country where efforts are now being made to improve access to treatment.

**Why Was This Study Done?**

The cost-effectiveness of HAART has been studied in developed countries, but developing countries also need to know how much it is going to cost their health services if they introduce HAART, and whether there will be financial savings because of switching to a more effective treatment.

**What Did the Researchers Do and Find?**

During the study period (January 1995 to 31 December 2000), HAART was not available in the publicly funded South African health-care sector. The study, funded by the drug manufacturer Bristol-Myers Squibb, took place in HIV clinics affiliated with the University of Cape Town. The researchers compared the cost of services for 292 patients who were given HAART with the costs for a comparison group (with the same number of patients) who were not given any antiretroviral drugs. Twenty-seven patients in each group had AIDS; the others were HIV-infected but did not have AIDS. The researchers calculated costs per patient year (PPY) and per life-year gained (LYG), i.e., the total cost divided by the number of extra years the treated patients lived. Calculations were done separately for patients with AIDS and those without AIDS. Patients on HAART required fewer hospital admissions. Depending on how long the patient survived and the price of antiretrovirals, it cost less to treat the HAART patients with AIDS. For this group, the cost saving ranged from $219 to $2,116 (in U.S. dollars). For patients without AIDS, the cost of treatment (ranging from $597 to $1,772) was, by the South African standard of cost of living, affordable. However, it is expected that South Africa will soon be able to manufacture antiretroviral drugs locally and more cheaply. This would increase the amount saved by introducing HAART.

**What Does This Mean?**

HAART seems to be a more cost-effective way for South African hospitals to treat HIV infection than simply waiting for patients to come to hospital and then dealing with their symptoms. However, it should be noted that when a person is infected with HIV and becomes ill or dies from AIDS, it is not only hospitals that face costs. The patient, their family, and the country suffer financially. Effective treatment might also lower these “indirect” costs, but this was not an issue examined in this research.

**Where Can I Find More Information Online?**

For a comprehensive source of information on HIV/AIDS:

http://www.thebody.com

The site also includes a useful section on HAART:

http://www.thebody.com/Forums/AIDS/Infections/Archive/NewMedications/Q12178.html

For information about the global AIDS situation and the position in different countries:

http://www.unaids.org.

## Abbreviations

ART: antiretroviral therapy
CI: confidence interval
HAART: highly active antiretroviral therapy
IQR: interquartile range
LYG: life-year gained
OR: odds ratio
PPY: per patient-year
WHO: World Health Organization

## References

[pmed-0030004-b001] UNAIDS (2004). HIV/AIDS epidemic in South Africa.

[pmed-0030004-b002] Dorrington R, Bourne D, Bradshaw D, Laubscher R, Timaeus I (2001). The impact of HIV/AIDS on adult mortality in South Africa. Technical report for the South African Medical Research Council.

[pmed-0030004-b003] Floyd K, Reid RA, Wilkinson D, Gilks C (1999). Admission trends in a rural South African Hospital during early years of the HIV epidemic. JAMA.

[pmed-0030004-b004] Hensher M (2001). Health sector impacts of HIV/AIDS. Key issues for planning.

[pmed-0030004-b005] Abt Associates (2001). Impending catastrophe revisited: An update on the HIV/AIDS epidemic in South Africa.

[pmed-0030004-b006] Freedberg KA, Losina E, Weinstein MC, Paltiel AD, Cohen CJ (2001). The cost effectiveness of combination antiretroviral therapy for HIV disease. N Engl J Med.

[pmed-0030004-b007] Sendi PP, Bucher HC, Harr T, Craig BA, Schwietert M (1999). Cost-effectiveness of highly antiretroviral therapy in HIV-infected patients: SWISS HIV cohort study. AIDS.

[pmed-0030004-b008] Moore R (2000). Cost-effectiveness of combination HIV therapy: 3 years later. Pharmacoeconomics.

[pmed-0030004-b009] Miners AH, Sabin CA, Trueman P, Youle M, Mocroft A (2001). Assessing the cost-effectiveness of HAART for adults with HIV in England. HIV Med.

[pmed-0030004-b010] Le Pen C, Rozenbaum W, Downs A, Maurel F, Lilliu H (2001). Effect of HAART on health status and hospital costs of severe HIV-infected patients: a modeling approach. HIV Clin Trials.

[pmed-0030004-b011] Beck EJ, Kennelly J, McKevitt C, Whitaker L, Wadsworth J (1994). Changing use of hospital services and costs at a London AIDS referral centre, 1983–1989. AIDS.

[pmed-0030004-b012] Beck EJ, Mandalia S, Williams I, Power A, Newson R for the NPMS Steering Group (1999) Decreased morbidity and use of hospital services in English HIV infected individuals with increased uptake of anti-retroviral therapy 1996–1997. AIDS.

[pmed-0030004-b013] Stoll M, Class C, Scuttle E, Graf, Von Der Sculenburg JM, Schmidt RE (2002). Direct costs for the treatment of HIV-infection in a German cohort after introduction of HAART. Eur J Med Res.

[pmed-0030004-b014] Torres RA, Barr M (1997). Impact of combination therapy for HIV infection on inpatient census. N Engl J Med.

[pmed-0030004-b015] Bozzette SA, Joyce G, McCaffrey DF, Leibowitz AA, Morton SC (2001). Expenditures for the care of HIV-infected patients in the era of highly active antiretroviral therapy. N Engl J Med.

[pmed-0030004-b016] Beck EJ, Tolley K, Power A, Mandalia S, Rutter P for the NPMS Steering Group (1998) Use and cost of HIV service provision in England. Pharmacoeconomics.

[pmed-0030004-b017] Beck EJ, Whitaker L, Kennelly J, McKevitt C, Wadsworth J (1994). Changing presentation and survival, service utilization and costs for AIDS patients: insights from a London referral centre. AIDS.

[pmed-0030004-b018] Cullinan K (2002). South Africa takes first steps to provide antiretrovirals. Bull World Health Organ.

[pmed-0030004-b019] Medecins Sans Frontiers (2005). http://www.accessmed-msf.org/documents/untanglingtheweb%207.pdf.

[pmed-0030004-b020] UNAIDS (2003). Access to treatment and care. Fact sheet 2003. UNAIDS, 2003.

[pmed-0030004-b021] Department of Health (2005). ARV drug tender awarded.

[pmed-0030004-b022] Badri M, Bekker LG, Orrell C, Pitt J, Cilliers F (2004). Initiating highly active antiretroviral therapy in sub-Saharan Africa: An assessment of the revised World Health Organization scaling-up guidelines. AIDS.

[pmed-0030004-b023] Badri M, Wilson D, Wood R (2002). Effect of highly active antiretroviral therapy on incidence of tuberculosis in South Africa: A cohort study. Lancet.

[pmed-0030004-b024] World Health Organization (1990). Acquired immunodeficiency syndrome (AIDS). Interim proposal for a WHO staging system for HIV infection and disease. Wkly Epidemiol Rec.

[pmed-0030004-b025] SAS Institute (2000). SAS OnlineDoc, version 8.02 [computer program].

[pmed-0030004-b026] Cape Metropolitan Council (1997). Levels of living in the Cape Metropolitan area, 2nd ed.

[pmed-0030004-b027] Beck EJ, Mandalia S, Gaudreault M, Brewer C, Zowall H (2004). The cost-effectiveness of highly active antiretroviral therapy, Canada 1991–2001. AIDS.

[pmed-0030004-b028] Beck EJ, Miners AH, Gazzard B, Johnson M, Miles A (2001). Effectiveness and efficiency in the delivery of HIV services: Economic and related considerations. The effective management of HIV disease.

[pmed-0030004-b029] Drummond MF, O'Brien B, Stoddart GL, Torrance GW (1997). Methods for the economic evaluation of health care programmes, 2nd ed.

[pmed-0030004-b030] Beck EJ, Miners A, Tolley K (2001). The cost of HIV treatment and care. A global review. Pharmacoeconomics.

[pmed-0030004-b031] Govender V, McIntyre D, Grimwood A, Maartens G (2000). The costs and perceived quality of care for people living with HIV/AIDS in the Western Cape Province in South Africa. Small-Applied Research No. 14.

[pmed-0030004-b032] Statistics South Africa (2004). Historical PPI key indicators.

[pmed-0030004-b033] US Federal Reserve Board (2005). Foreign exchange rates. South Africa historical rates (annual).

[pmed-0030004-b034] World Health Organization (2004). Scaling up antiretroviral therapy in resource-limited settings: Treatment guidelines for a public health approach. 2003 revision.

[pmed-0030004-b035] Parsonage M, Neuberger H (1992). Discounting of health benefits. Health Economics.

[pmed-0030004-b036] Ngalula J, Urassa M, Mwaluko G, Isingo R, Boerma T (2002). Health service use and household expenditure during terminal illness due to AIDS in rural Tanzania. Trop Med Int Health.

[pmed-0030004-b037] Arthur G, Bhatt SM, Muhindi D, Achiya GA, Kariuki SM (2000). The changing impact of HIV/AIDS on Kenyatta National Hospital, Nairobi from 1988/89 through 1992 to 1997. AIDS.

[pmed-0030004-b038] Buve A (1997). AIDS and hospital bed occupancy: An overview. Trop Med Intern Health.

[pmed-0030004-b039] Tembo G, Friesan H, Asiimwe-Okiror G, Moser R, Naamara W (1994). Bed occupancy due to HIV/AIDS in an urban hospital medical ward in Uganda. AIDS.

[pmed-0030004-b040] Nunn P, Gathua S, Kibuga D, Binge R, Brindle R (1993). The impact of HIV on resource utilization by patients with tuberculosis in a tertiary referral hospital, Nairobi, Kenya. Tuber Lung Dis.

[pmed-0030004-b041] Beck EJ, Mandalia S, Power A, Easterbrook P, Pozniak A (2000). Reduced HIV disease progression and mortality due to CART in English NPMS-HHC clinics [abstract]. XIII International AIDS Conference; 2000 9 July–14 July; Durban, South Africa.

[pmed-0030004-b042] Hore R, Cross S, Whiteside A (1993). The medical costs of AIDS in Zimbabwe. Facing up to AIDS: The socio-economic impact in southern Africa.

[pmed-0030004-b043] Harling G, Wood R, Beck EJ (2005). A review of the efficiency of interventions in HIV infection, 1994–2004. Dis Manage Health Outcomes.

[pmed-0030004-b044] Garber AM, Culyer AJ, Newhouse JP (2000). Advances in cost-effectiveness analysis of health interventions. Handbook of health economics, volume 1A.

[pmed-0030004-b045] Bureau of Public Affairs, U.S. Department of State (2005). http://www.state.gov/r/pa/ei/bgn/2898.htm.

[pmed-0030004-b046] Harries AD, Nyangulu DS, Hargreaves NJ, Kaluwa O, Salaniponi FM (2002). Preventing antiretroviral anarchy in Africa. Lancet.

[pmed-0030004-b047] Orrell C, Bangsberg DR, Badri M, Wood R (2003). Adherence is not a barrier to successful antiretroviral therapy in South Africa. AIDS.

[pmed-0030004-b048] Laurent C, Diakhate N, Gueye NF, Toure MA, Sow PS (2002). The Senegalese government's highly antiretroviral therapy initiative: An 18-month follow-up study. AIDS.

[pmed-0030004-b049] Weidle PJ, Malamba S, Mwebaze R, Sozi C, Rukundo G (2002). Assessment of a pilot antiretroviral therapy programme in Uganda: Patients' response, survival, and drug resistance. Lancet.

[pmed-0030004-b050] Geffen N, Natrass N, Raubenheimer C (2003). The cost of HIV prevention and treatment interventions in South Africa. CSSR working paper no 28.

[pmed-0030004-b051] Boulle A, Kenyon C, Skordis J, Wood R (2002). Exploring of the costs of a limited public sector antiretroviral treatment program in South Africa. S Afr Med J.

[pmed-0030004-b052] Bell C, Devarajan S, Gersbach H (2003). http://www1.worldbank.org/hiv_aids/docs/BeDeGe_BP_total2.pdf.

